# Tuning the Site-to-Site Interaction of Heteronuclear Diatom Catalysts MoTM/C_2_N (TM = 3d Transition Metal) for Electrochemical Ammonia Synthesis

**DOI:** 10.3390/molecules28104003

**Published:** 2023-05-10

**Authors:** Xiaoli Yang, Ping An, Ruiying Wang, Jianfeng Jia

**Affiliations:** 1 Key Laboratory of Magnetic Molecules and Magnetic Information Materials (Ministry of Education), School of Chemistry and Material Science, Shanxi Normal University, Taiyuan 030031, China; 2 Department of Pharmacy, Changzhi Medical College, Changzhi 046000, China

**Keywords:** electrochemical ammonia synthesis, heteronuclear diatom catalysts, density functional theory

## Abstract

Ammonia (NH_3_) synthesis is one of the most important catalytic reactions in energy and chemical fertilizer production, which is of great significance to the sustainable development of society and the economy. The electrochemical nitrogen reduction reaction (eNRR), especially when driven by renewable energy, is generally regarded as an energy-efficient and sustainable process to synthesize NH_3_ in ambient conditions. However, the performance of the electrocatalyst is far below expectations, with the lack of a high-efficiency catalyst being the main obstacle. Herein, by means of comprehensive spin-polarized density functional theory (DFT) computations, the catalytic performance of MoTM/C_2_N (TM = 3d transition metal) for use in eNRR was systematically evaluated. Among the results, MoFe/C_2_N can be considered the most promising catalyst due to its having the lowest limiting potential (−0.26 V) and high selectivity in the context of eNRR. Compared with its homonuclear counterparts, MoMo/C_2_N and FeFe/C_2_N, MoFe/C_2_N can balance the first protonation step and the sixth protonation step synergistically, showing outstanding activity regarding eNRR. Our work not only opens a new door to advancing sustainable NH_3_ production by tailoring the active sites of heteronuclear diatom catalysts but also promotes the design and production of novel low-cost and efficient nanocatalysts.

## 1. Introduction

Ammonia (NH_3_) is one of the most productive inorganic compounds in the world, with an annual output of about 150 million tons. It is used for the manufacture of many important chemicals, particularly fertilizers [1]. At present, industrial NH_3_ synthesis still adopts a high-temperature and high-pressure Haber–Bosch process invented more than a century ago [2]. The Haber–Bosch method of ammonia synthesis has successfully altered the history of food production, fed explosive population growth, and laid the foundation for heterogeneous catalysis and chemical engineering as well. However, this synthetic method consumes 1–2% of the world’s energy supply and about 2% of the natural gas supply and also produces 1.44% of global carbon dioxide (CO_2_) emissions [1,3,4,5,6]. Therefore, it is necessary to find more sustainable and environmentally friendly alternatives as soon as possible to replace the energy- and carbon-intensive Haber–Bosch process [1,3,7,8]. Making full use of the abundant nitrogen (N_2_) in the Earth’s atmosphere and using water as the proton source, an electrochemical nitrogen reduction reaction (eNRR), especially when driven by renewable energy, is generally regarded as an energy-efficient and sustainable process to synthesize NH_3_ in ambient conditions [9,10]. However, the performance of the electrocatalyst is far below expectations, and the lack of a high-efficiency catalyst is the main obstacle to large-scale NH_3_ synthesis.

Ever since Zhang Tao and co-workers first proposed the concept of a single-atom catalyst (SAC) in 2011 [11], single-atom catalysts (SACs), considered a hot spot in the field of heterogeneous catalysis in recent years, have been widely and exhaustively studied for their high atom utilization rate and excellent catalytic performance. In the context of theory, many SACs, such as Fe-N_3_/graphene [12], W-C_3_/graphene [13], Ti-N_4_/graphene [14], Mo/BN [15], Mo/C_2_N [16], Ru/*g*-C_3_N_4_ [17], and single-B catalysts [18,19,20], were predicted to be high-performance eNRR catalysts. Experimental research shows that some SACs can suppress the hydrogen evolution reaction (HER) to some extent and, thus, are capable of improving Faradaic efficiency (FE) [21,22,23,24,25,26]. SACs contain only one metal center, while eNRR involves several reaction steps and intermediates. This contradiction makes it difficult for SACs to break the linear adsorption relationship of reaction intermediates and balance several reaction steps simultaneously. In order to solve this problem, a wise strategy is to introduce a second metal atom near the original single metal atom to form a diatomic active center, which helps to tune the adsorption properties of the target intermediate [27]. Such diatom catalysts (DACs) have more flexible active sites and synergistic interatomic interactions, which can maximize the potential of SACs for multistep reactions. In addition, DACs offer more possibilities than SACs, which also provide rich candidates for screening advanced catalysts [3,27,28,29].

In 2015, Baek et al. first fabricated a novel two-dimensional layered material of C_2_N with uniform hole distribution [30]. The unique porous structure provides ideal support for the metallic active center, while the high surface-to-volume ratio ensures the sufficient exposure of transition metal (TM) atoms to interact with the reactant molecules [31]. Therefore, C_2_N, as a two-dimensional base material loaded with transition metals or nonmetallic elements, has attracted more and more attention. More importantly, the well-ordered large holes in C_2_N offer the opportunity to anchor two TM atoms tightly together, which efficiently suppresses the diffusion and aggregation of TM atoms [32,33,34]. For example, Jiang et al. identified that Co_2_/C_2_N, Ni_2_/C_2_N, and Cu_2_/C_2_N exhibit high adsorption energies and extremely low dissociation barriers for O_2_ [32]. Zhou et al. designed a series of catalysts of single or double transition metal atoms anchored on a C_2_N monolayer and studied their catalytic performance regarding NRR. Among them, Mo_2_/C_2_N exhibits the best catalytic performance [34]. An’s research group investigated the synergy of a metallic NiCo dimer anchored on a C_2_N graphene matrix for promoting the CO_2_ reduction reaction. It was found that heterometallic NiCo/C_2_N outperforms homometallic Co_2_/C_2_N and Ni_2_/C_2_N in terms of catalyzing the CO_2_ reduction reaction toward CH_4_ formation, owing to synergy within the dimer [35].

So far, most research efforts have primarily focused on homonuclear DACs. However, in principle, heteronuclear DACs have more possibilities waiting for us to explore. Previous theoretical studies showed that the single-atom catalyst Mo/C_2_N [16] and the double-atom catalyst Mo_2_/C_2_N [34] offer good performance regarding eNRR. Considering the regular change of 3d elements from Sc to Ni (the number of 3d electrons gradually increases, the atomic radius gradually decreases, and the electronegativity gradually increases), we constructed a series of heteronuclear diatom catalysts MoTM/C_2_N (TM = 3d transition metal) to investigate the eNRR mechanism and performance. Through the regular tuning of 3d transition metals, it is expected that researchers will obtain better eNRR catalysts and reveal the essence of tuning. The results show that the heteronuclear diatom catalyst MoFe/C_2_N is the best eNRR catalyst and has the lowest limiting potential (−0.26 V), which is superior to its homonuclear counterparts, MoMo/C_2_N and FeFe/C_2_N. This work not only opens a new door to advancing sustainable NH_3_ production by tailoring the active sites of heteronuclear DACs but also promotes the design and production of novel low-cost and efficient nanocatalysts.

## 2. Results and Discussion

### 2.1. Geometric Structures, Stabilities, and Electronic Properties of the MoTM/C_2_N (TM = 3d Transition Metal)

**Figure 1:**
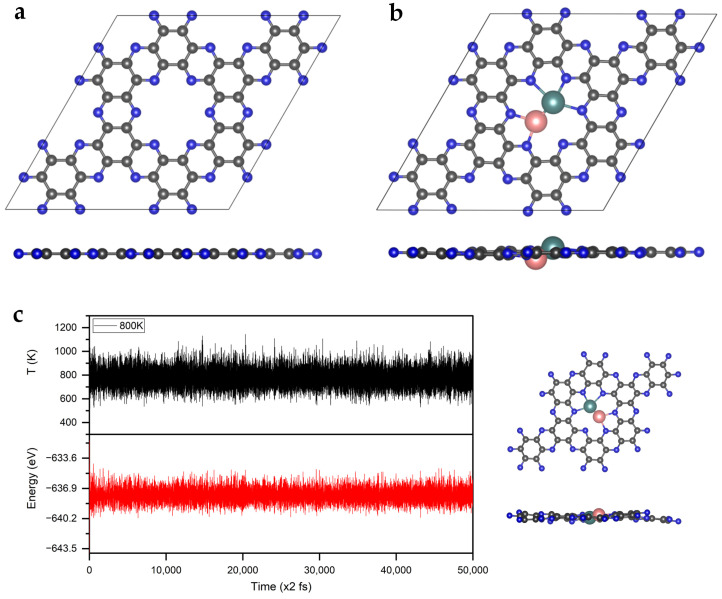
The optimized structures of (**a**) C_2_N and (**b**) MoFe/C_2_N. The C, N, Mo, and Fe atoms are labeled as gray, blue, teal, and pink balls, respectively. (**c**) Variations in temperature and energy, shown against the time for AIMD simulations of MoFe/C_2_N; the inserts show the top and side views of the snapshot of the atomic configuration. The simulation is run under 800 K for 100 ps, with a time step of 2 fs.

First, the geometric structure of the C_2_N monolayer was optimized and the lattice parameters were calculated to be *a* = *b* = 8.32 Å, which matches well with the experimental data and previous DFT reports (Figure 1a) [30,34]. In the C_2_N monolayer, benzene rings are bridged by pyrazine rings to form six-membered nitrogen holes, in which the distance between two opposite nitrogen atoms is 5.51 Å [33].

Then, we investigated the geometric structures and the stability of MoTM/C_2_N (TM = 3d transition metal). The optimized structures of MoTM/C_2_N are presented in Figure S1 and Table S1 in the Supplementary Materials. Clearly, the framework structure of C_2_N can be maintained well with the decoration of Mo and TM atoms. Except for the larger atomic radius of Sc, which slightly protrudes out of the C_2_N plane, all the other metal atoms can be embedded into the central cavity of C_2_N holes and form almost coplanar structures. The bond lengths of Mo-TM are in the range of 1.93 Å (for MoV/C_2_N) and 2.48 Å (for MoSc/C_2_N). Compared with the length of the metal bond in the corresponding bulk phase, the bond lengths of Mo-TM in the system of MoTM/C_2_N are shorter. Such interconnections enable the heteronuclear diatom to respond cooperatively to the adsorbate, with communicative structural self-adaption and electronic transformation, which induces a different catalytic performance from that of its single-atom counterparts [3,27,36]. The binding energy (*E*_b_) of MoTM and the Bader charge of anchored Mo and TM were calculated to examine structural stability, as shown in Table S1 in the Supplementary Materials. The smallest *E*_b_ of MoTM on C_2_N is −9.10 eV, suggesting strong coupling and good stability. Meanwhile, the Bader charge analysis showed that the atomic charge of the anchored TM atoms (from Sc to Ni) decreased gradually from +2.32 to +0.43, which is due to the increasing electronegativity of TM atoms (from Sc to Ni). Conversely, the atomic charge of anchored Mo atoms gradually increased.

In order to evaluate the thermal stability of MoFe/C_2_N, we simulated MoFe/C_2_N using the ab initio molecular dynamics (AIMD) method. As shown in Figure 1c, the energy fluctuation oscillates around the equilibrium state according to time evolution. Even at 800 K, the structure of MoFe/C_2_N remained stable after the 100 ps simulation, showing high thermal stability. This high stability stems from the strong bonding between Mo and Fe, as well as between the MoFe and N atoms of the C_2_N substrate. In addition, the AIMD results also show that the anchored Mo and Fe atoms cannot escape from the holes in the substrate, thus excluding the problems of diffusion and agglomeration.

### 2.2. Adsorption of N_2_

**Figure 2:**
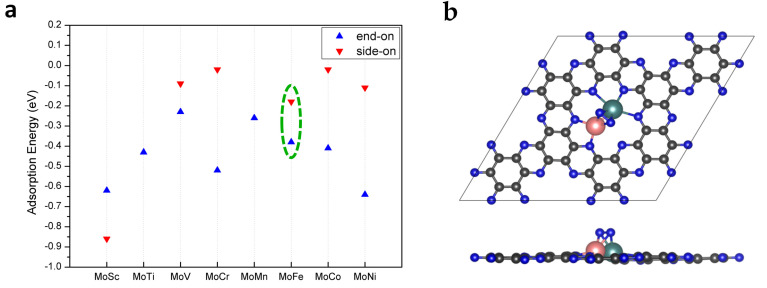
(**a**) The adsorption energies of N_2_ on MoTM/C_2_N, with the end-on and side-on modes. (**b**) Adsorption of N_2_ on the bimetallic site of MoFe/C_2_N in the side-on mode. The C, N, Mo, and Fe atoms are labeled as gray, blue, teal, and pink balls, respectively.

It is well known that the adsorption of reactants on the catalyst surface is the key step to initializing a reaction. In order to comprehensively study the adsorption of N_2_ on the catalyst’s surface, we considered seven possible adsorption configurations, in which N_2_ is adsorbed on the top site of a single metal or bimetallic bridge site via the end-on or side-on mode, as shown in Figure S2 in the Supplementary Materials. After full structural relaxation, the most favorable adsorption configurations of N_2_ on MoTM/C_2_N are presented in Figure S3 in the Supplementary Materials. The adsorption energies of N_2_ were calculated and are displayed in Figure 2a.

From the point of view of adsorption energy, N_2_ tends to be adsorbed onto MoTM/C_2_N (except MoSc/C_2_N) via the end-on mode. Because of the geometric constraint that the Sc atom protrudes out of the catalyst surface, N_2_ is preferably adsorbed on MoSc/C_2_N via the side-on mode instead of the end-on mode. It can be clearly seen from Figure S3 in the Supplementary Materials that the site where N_2_ is adsorbed onto MoTM/C_2_N via the end-on mode is the top site of the Mo atom. However, there are two kinds of sites where N_2_ is adsorbed onto the catalyst surface via the side-on mode: one (MoTM/C_2_N, TM = Cr and Ni) is the top site of the Mo atom, and the other (MoTM/C_2_N, TM = Sc, V, Fe, and Co) is a bimetallic bridge site. The side-on adsorption configuration of N_2_ on MoFe/C_2_N as a representative is displayed in Figure 2b. According to previous studies [27], the moderate adsorption strengths and small differences in N_2_ adsorption energies between side-on and end-on adsorption configurations suggest that MoFe/C_2_N may be a good eNRR catalyst.

Besides the adsorption energy, the Bader charge, charge density difference, and bond length of the adsorbed N_2_ were also measured and are shown in Figure S3 in the Supplementary Materials. Charge density difference plots indicate that the charge transfer between the adsorbed N_2_ and the anchored MoTM is bidirectional, and charge accumulation and depletion can be observed on both sides. This phenomenon is in perfect accordance with the “push-pull” hypothesis, in which the anchored MoTM can “push” the d electrons into the antibonding orbitals of N_2_ and simultaneously “pull” the lone-pair electrons from N_2_. More importantly, we found that there is a good correlation between the N-N bond length and the Bader charge of N_2_; that is, the more negative the Bader charge of N_2_ is, the longer the N-N bond length is. For the side-on adsorption configurations of N_2_ on the bimetallic bridge site of MoTM/C_2_N (TM = Sc, V, Fe and Co), the Bader charge of N_2_ decreases to between −0.82 |*e*| and −1.05 |*e*|, with their N-N bond lengths obviously elongated to 1.213~1.293 Å (1.12 Å in the free gas phase). In the case of the side-on adsorption configurations of N_2_ on the top site of the Mo atom of the MoTM/C_2_N (TM = Cr and Ni), the Bader charge of N_2_ decreases to between −0.46 |*e*| and −0.49 |*e*|, with their N-N bond lengths elongated to 1.176–1.178 Å. In comparison, the end-on adsorption configurations of N_2_ on the top site of the Mo-atom of MoTM/C_2_N, the Bader charge of N_2_ decreases to between −0.34 |*e*| and 0.42 |*e*| with their N-N bond lengths slightly elongated to 1.138~1.143 Å. As is consistent with previous reports [27,37,38], this means that the activation of adsorbed N_2_ with the side-on mode is stronger than that with the end-on mode.

### 2.3. Catalytic Activity for eNRR

**Figure 3:**
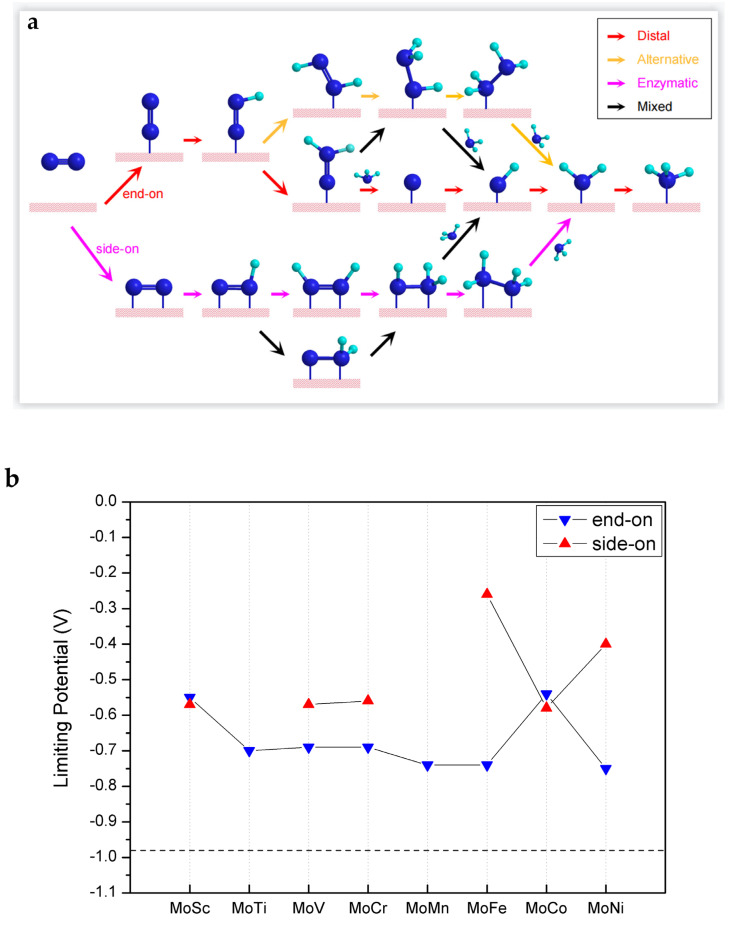

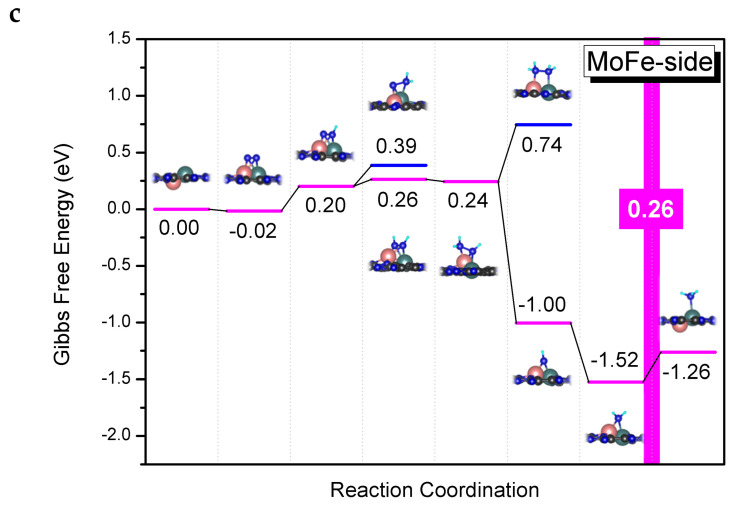
(**a**) Schematic illustration of the possible reaction mechanisms during the N_2_ reduction reaction. (**b**) Limiting potentials for eNRR on MoTM/C_2_N. (**c**) Gibbs free energy diagrams for eNRR on MoFe/C_2_N in the side-on mode.

Next, we move to the assessment of the catalytic performance of MoTM/C_2_N for the reduction of N_2_ into NH_3_. Four possible associative NRR catalytic mechanisms, namely, distal, alternative, enzymatic, and mixed mechanisms, are possible with MoTM/C_2_N catalysts, as is schematically illustrated in Figure 3a. In the case of end-on adsorption, the conversion of N_2_ to NH_3_ follows a distal or alternative or mixed mechanism. In the case of side-on adsorption, the conversion of N_2_ to NH_3_ follows either an enzymatic or mixed mechanism.

In principle, the intrinsic activity of electrocatalysts can be evaluated by limiting potential (*U*_L_) or overpotential. Considering that overpotential can be affected by electrolyte conditions, we choose the limiting potential (*U*_L_) as the evaluation standard in this paper. Among all the protonation steps, the protonation step with the largest positive Gibbs free energy difference is defined as the potential-determination step (PDS), while the lowest negative potential that promotes PDS to become exothermic is defined as the limiting potential (*U*_L_ = −Δ*G*_PDS_/e). As shown in Figure 3b, the *U*_L_ of all catalysts MoTM/C_2_N (TM = 3d transition metal) were marked, and the catalytic activity of the widely reported Ru (0001) stepped surface (−0.98 V) was taken as a benchmark. As can be seen, the catalytic activity of all candidate catalysts of MoTM/C_2_N is superior to that of the Ru (0001) stepped surface.

Detailed Gibbs free energy diagrams of eNRR on MoTM/C_2_N are given in Figures S4–S11. In the case of end-on adsorption, the PDS of eNRR is generally the first protonation step, that is, the generation of *NNH (where * stands for end-on adsorption), owing to the relatively low charge transfer from the substrate to N_2_. In the case of side-on adsorption, the PDS of the eNRR is the sixth protonation step, that is, the generation of the second ammonia, owing to the effective activation of N_2_. Among them, the limiting potential of eNRR on MoFe/C_2_N with side-on adsorption is the lowest (−0.26 V). Therefore, we now focus on analyzing the reaction path of eNRR on MoFe/C_2_N.

The Gibbs free energy diagrams of eNRR on the MoFe/C_2_N catalyst with side-on adsorption are presented in Figure 3c. In the reduction process, **NNH (where ** stands for side-on adsorption) is formed by the interaction between the **N_2_ and H^+^/e^−^ pair, and the first protonation step (**N_2_ + H^+^ + e^−^ → **NNH) is 0.22 eV uphill in the free energy profile. When **NNH is protonated further, the H^+^/e^−^ pair can continue to attack the same N atom to form **NNH_2_ or attack another N atom to form **NHNH. Comparing the Gibbs free energy changes of these two elementary steps, we found that it prefers to go through a step (**NNH + H^+^ + e^−^ → **NHNH) with a Gibbs free energy uphill of 0.06 eV. Subsequently, the **NHNH can be protonated to **NH_2_NH easily, and this step (**NHNH + H^+^ + e^−^ → **NH_2_NH) is exothermic at 0.02 eV. Once the **NH_2_NH is formed, the first NH_3_ molecule can be readily desorbed from the catalyst surface, leaving *NH (the symbol * here only represents the adsorption state) on the catalyst surface and releasing 1.24 eV of heat. The fifth protonation step (*NH + H^+^ + e^−^ → *NH_2_) is exothermic at 0.52 eV. The sixth protonation step (*NH_2_ + H^+^ + e^−^ → *NH_3_) is endothermic at 0.26 eV. Among all these elementary steps, the sixth protonation step has the largest positive Gibbs free energy difference of 0.26 eV; therefore, it is considered the PDS. To sum up, the eNRR reaction on MoFe/C_2_N follows the mixed mechanism, and the *U*_L_ is −0.26 V.

**Figure 4:**
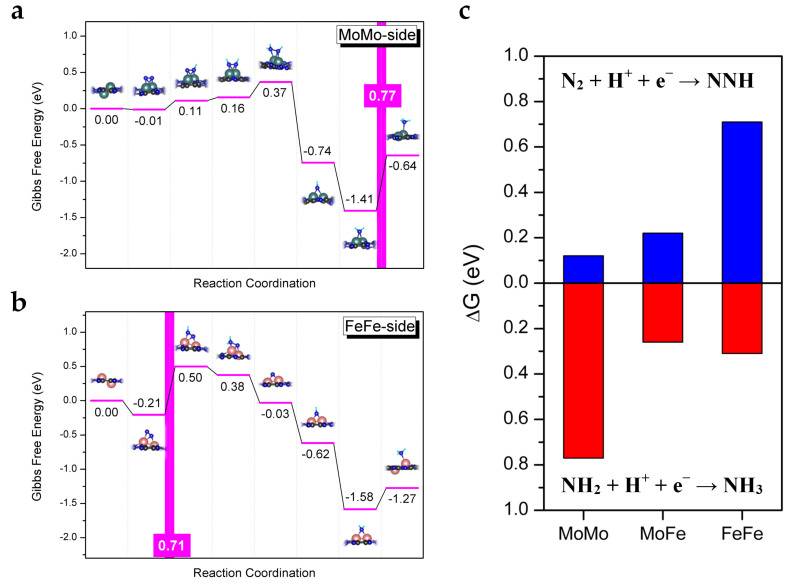
(**a**,**b**) Gibbs free energy diagrams of eNRR with N_2_ side-on adsorption on MoMo/C_2_N and FeFe/C_2_N, respectively. (**c**) Comparison diagram of Gibbs free energy change of the first protonation step and the sixth protonation step on the MoMo/C_2_N, MoFe/C_2_N, and FeFe/C_2_N.

For comparison, we also investigated the catalytic performance of the homonuclear diatomic catalysts, MoMo/C_2_N and FeFe/C_2_N, with N_2_ side-on adsorption for eNRR, as shown in Figure 4. Note that the sixth protonation step (*NH_2_ + H^+^ + e^−^ → *NH_3_) on MoMo/C_2_N is the PDS with a maximum free energy change of 0.77 eV, and the first protonation step (**N_2_ + H^+^ + e^−^ → **NNH) on FeFe/C_2_N is the PDS with a maximum free energy change of 0.71 eV. As mentioned in the literature [38], the hydrogenation of *NH_2_ species to *NH_3_ is identified as the PDS on Mo-based catalysts, while the formation step of * NNH species is identified as the PDS on Fe-based catalysts. However, the energy changes of the first protonation step and the sixth protonation step on the heteronuclear diatomic catalyst MoFe/C_2_N are both small (0.22 eV and 0.26 eV, respectively). Notably, compared with MoMo/C_2_N and FeFe/C_2_N, MoFe/C_2_N can balance the first protonation step and the sixth protonation step synergistically, showing outstanding activity for eNRR. From this perspective, the heteronuclear diatomic catalyst is more effective than its homonuclear counterparts. Moreover, except for their high catalytic activity, MoFe-based catalysts also possess other merits, such as nontoxicity and a low price [39,40,41,42].

### 2.4. Selectivity of MoFe/C_2_N

**Figure 5:**
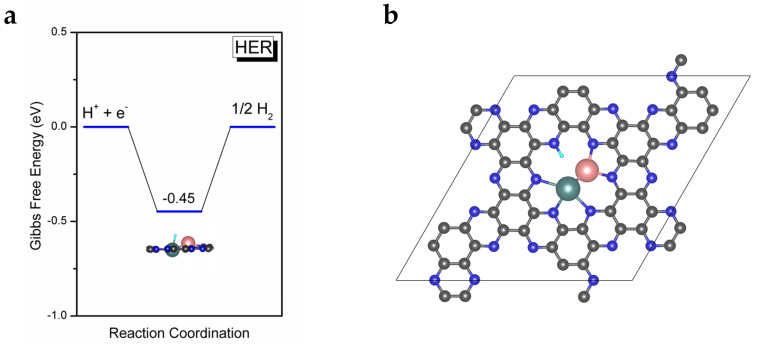
(**a**) Gibbs free energy diagram of HER on MoFe/C_2_N. (**b**) The *H adsorption on the N site of MoFe/C_2_N.

As we all know, the hydrogen evolution reaction (HER) is the major competing reaction in eNRR and significantly affects the Faradaic efficiency of eNRR. It is necessary for an excellent eNRR catalyst to effectively suppress HER. In order to test the selectivity of MoFe/C_2_N for eNRR, we calculated the adsorption free energy of H on MoFe/C_2_N and used it to estimate the overpotential of HER. The *H structure of the adsorption site and corresponding free energy diagram are depicted in Figure 5a. The free energy barrier (0.45 eV) is considerably larger than the PDS barrier for eNRR (0.26 eV) on heteronuclear MoFe/C_2_N. Thus, MoFe/C_2_N exhibits an excellent suppression effect on HER during eNRR. In summary, as we expected, the heteronuclear diatom catalyst MoFe/C_2_N exhibits superior catalytic activity for eNRR, with a limiting potential of −0.26 V, and possesses high selectivity toward eNRR.

Because the catalyst MoFe/C_2_N itself contains the N element, another problem to be solved is to exclude the nitrogen source of produced NH_3_ from the nitrogen-containing catalyst, that is, to judge whether the well-known Mars–van Krevelen (MvK) pathway [43,44] will occur on MoFe/C_2_N. It is worth noting that in the MvK pathway, a lattice N atom of the catalyst is reduced to NH_3_; the catalyst is subsequently regenerated with gaseous N_2_, in which the adsorption of *H species on the N site of the catalyst is the first and most critical step. In order to solve this problem, we examined the *H adsorption on the N site of the MoFe/C_2_N (Figure 5b). The result showed that the *E*_ad_ of *H on the N site of MoFe/C_2_N is almost zero. Thus, the N site of MoFe/C_2_N is unlikely to be the active site for eNRR, implying that the reactant N_2_ molecule is the only N source for the NH_3_ product.

### 2.5. Electronic Structures

**Figure 6:**
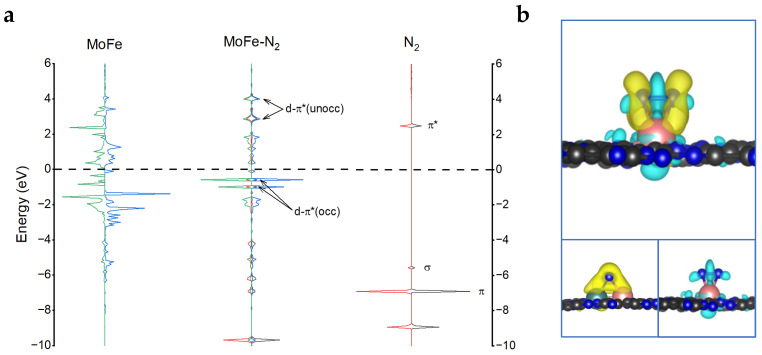
(**a**) Projected electronic densities of states (pDOS) of MoFe (d orbitals) within the MoFe/C_2_N and N_2_ gas molecule before and after side-on adsorption. (**b**) Charge density differences in N_2_ adsorption on MoFe/C_2_N with the side-on mode. The charge accumulation and depletion are depicted in yellow and cyan, respectively. The isosurface value is 0.003 e/Å^3^.

To reveal the origin of MoFe/C_2_N as an efficient catalyst for eNRR, the electronic structure was investigated. As can be seen from Figure 6a, before N_2_ adsorption on MoFe/C_2_N, the d orbital hybridized peaks between Mo and Fe are observed, which means that the bond of Mo and Fe is formed in the C_2_N substrate. After N_2_ adsorption with the side-on mode, the energy levels of MoFe d orbitals and N_2_ π* orbitals are well matched, leading to an anti-bonding orbital d-π*(unocc) above the Fermi level (located at 2.88 eV and 4.01 eV) and a bonding orbital d-π*(occ) below the Fermi level (located at −0.98 eV and −0.58 eV). This strong interaction between N_2_ and MoFe metal atoms leads to the formation of N-Mo and N-Fe, as well as the weakening of the N-N bond, which facilitates the subsequent reduction reaction of N_2_. Meanwhile, this is also clearly confirmed by the charge density difference diagram (Figure 6b). The charge accumulation (shown in yellow) is mainly distributed between N and Mo, as well as between N and Fe, indicating the formation of N-Mo bonds and N-Fe bonds. In contrast, charge depletion (shown in cyan) is mainly distributed in the N-N bond, indicating that the N-N bond is weakened.

## 3. Computational Methods

In this work, all the computations were carried out using the spin-polarized DFT method, which includes van der Waals (vdW) corrections, as implemented in the Vienna ab-initio simulation package (VASP) [45,46,47]. The projector-augmented-wave (PAW) method and the generalized gradient approximation (GGA) with a Perdew–Burke–Ernzerhof (PBE) exchange-correlation function were used [48,49,50]. Grimme’s semiempirical DFT-D3 scheme of dispersion correction was employed to account for the weak interactions [51,52]. Lattice parameters were fully relaxed, and the crystal geometry was fully optimized before electron structure and total energy calculations. A plane-wave cutoff energy of 600 eV was set to evaluate the core-electron interaction, and the total energy and force converged within 10^−6^ eV and 0.02 eV·Å^−1^, respectively. The convergence standard for the calculation of the vibrational frequency was set at 10^−7^ eV. The integration of the Brillouin zones was sampled with 2 × 2 × 1 Monkhorst–Pack meshes [53]. In order to prevent any artificial interaction between periodically repeated images, a vacuum space of 15 Å was inserted along the *z* direction to the monolayers. The atomic charge was computed using Bader’s charge population analysis [54]. To evaluate the stability of the catalysts, ab initio molecular dynamics (AIMD) simulations were performed in an NVT ensemble. The AIMD simulations lasted 100 ps, with a time step of 2.0 fs, and the temperature was controlled using the Nosé–Hoover method [55].

The binding energy (*E*_b_) of the MoTM atoms was computed from the following equation:

*E*_b_ = *E*_MoTM/C2N_ − *E*_C2N_ − *E*_Mo_ − *E*_TM_ (1)

where *E*_MoTM/C2N_, *E*_C2N_, *E*_Mo_, and *E*_TM_ represent the total energy of MoTM/C_2_N, the energy of the substrate C_2_N, the energy of the gas-phase Mo atom, and the energy of the gas-phase TM atom, respectively.

The adsorption energy (*E*_ad_) of the adsorbates was computed according to the following equation:

*E*_ad_ = *E*_adsorbate_ + *E*_MoTM/C2N_ − *E*_MoTM/C2N_ − *E*_adsorbate_ (2)

where *E*_adsorbate_ + *E*_MoTM/C2N_, *E*_MoTM/C2N_, and *E*_adsorbate_ represent the total energy of the catalyst MoTM/C_2_N and the adsorbate, the energy of the catalyst MoTM/C_2_N, and the energy of free adsorbate, respectively. According to this definition, the larger the negative value of *E*_ad_ is, the more stable the adsorption state is.

The computational hydrogen electrode (CHE) model proposed by Nørskov et al. was used to compute the free energy change of each elementary step for the N_2_ reduction reaction [56]. According to the CHE model, the chemical potential of the H^+^/e^−^ pair is equal to half that of H_2_ with the standard conditions of pH = 0 and *p*(H_2_) = 1 bar: $\mu_{H^{+}}+\mu_{e^{-}}=\frac{1}{2}\mu_{H_{2}}$. The free energy change (Δ*G*) of each protonation step was computed according to the following equation:

Δ*G* = Δ*E* + Δ*E*_ZPE_ − *T*Δ*S* + Δ*G*_U_ + Δ*G*_pH_ (3)

where Δ*E* is the reaction energy obtained from DFT computations, Δ*E*_ZPE_ is the change of zero-point energy, T is the temperature (298.15 K), and Δ*S* is the entropy change. The entropies of the gas phase were taken from the NIST database. The entropies and zero-point energy of the adsorbates were calculated based on their vibrational frequencies; the entropy can be determined as follows [57,58]:

(4) $$S(T)=\sum_{i=1}^{3N}\left[-R\ln\left(1-e^{-\frac{h\nu_{i}}{k_{B}T}}\right)+\frac{N_{A}h\nu_{i}}{T}\frac{e^{-h\nu_{i}/k_{B}T}}{1-e^{-h\nu_{i}/k_{B}T}}\right]$$

where *R* is the universal gas constant, *h* is Planck’s constant, *k*_B_ represents Boltzmann’s constant, *N*_A_ indicates Avogadro’s number, *ν_i_* represents the frequency of the normal mode, and *N* is the number of adsorbed atoms. Δ*G_U_* could be computed using Δ*G_U_* = −*eU*, where *e* represents the charge transferred and *U* represents the potential at the electrode. Δ*G*_pH_ is the correction of the H^+^ free energy by the concentration and can be expressed as Δ*G*_pH_ = *k*_B_*T* ln 10 × pH.

## 4. Conclusions

In order to obtain more efficient electrocatalysts with higher Faradaic efficiency, we have constructed a series of heteronuclear diatomic catalysts of MoTM/C_2_N (TM = 3d transition metal) for eNRR by tuning the interaction between Mo and TM. Then, the stability, activity, and selectivity of MoTM/C_2_N catalysts have been comprehensively evaluated using the spin-polarized DFT method. Among them, MoFe/C_2_N has been identified as the most promising catalyst, due to its low value of *U*_L_ (−0.26 V) and high selectivity toward the eNRR. In order to reach a deeper understanding, we have compared MoFe/C_2_N with its homonuclear counterparts, MoMo/C_2_N and FeFe/C_2_N. The results show that MoFe/C_2_N can balance the first protonation step and the sixth protonation step synergistically, which is the origin of its excellent activity. We hope that our work will inspire more experimental and theoretical studies to further explore the potential of heteronuclear DACs in other chemical reactions.

## Data Availability

Not applicable.

## Supplementary Materials

The following supporting information can be downloaded at: https://www.mdpi.com/article/10.3390/molecules28104003/s1, Figure S1: Optimized structures of the most stable MoTM/C_2_N (TM = 3d transition metal). The C, N and Mo atoms are labeled as gray, blue and teal balls, respectively. Figure S2: Possible adsorption configurations diagram of N_2_ on MoTM/C_2_N catalysts (take MoTi/C_2_N as an example). The C, N, Mo and Ti atoms are labeled as gray, blue, teal and red balls, respectively. Figure S3: Optimized adsorption configurations and charge density differences of N_2_ chemisorbed on MoTM/C_2_N (TM = 3d transition metal). The charge accumulation and depletion were depicted by yellow and cyan, respectively. The isosurface value is 0.003 e/Å^3.^ Figure S4: Gibbs free energy diagrams for NRR on MoSc/C_2_N. Figure S5: Gibbs free energy diagram for NRR on MoTi/C_2_N. Figure S6: Gibbs free energy diagrams for NRR on MoV/C_2_N. Figure S7: Gibbs free energy diagrams for NRR on MoCr/C_2_N. Figure S8: Gibbs free energy diagram for NRR on MoMn/C_2_N. Figure S9: Gibbs free energy diagrams for NRR on MoFe/C_2_N. Figure S10: Gibbs free energy diagrams for NRR on MoCo/C_2_N. Figure S11: Gibbs free energy diagrams for NRR on MoNi/C_2_N. Figure S12: Gibbs free energy diagrams for NRR on MoMo/C_2_N and FeFe/C_2_N, respectively. Figure S13: Gibbs free energy diagram of HER on MoFe/C_2_N. Figure S14: The *H adsorption on the N site of MoFe/C_2_N. Table S1: The bond length of Mo-TM (d_Mo-TM_), the binding energy (*E*_b_) of MoTM and the atomic charge of anchored Mo and TM in the system of MoTM/C_2_N (TM = 3d transition metal), and the bond length of TM-TM in their bulk phase (D_TM-TM_).
