# Supplementary material for: Tuning the Site-to-Site Interaction of Heteronuclear Diatom Catalysts MoTM/C2N (TM = 3d Transition Metal) for Electrochemical Ammonia Synthesis
Source: Molecules. 2023 May 10;28(10):4003. doi: 10.3390/molecules28104003 (PMC10222264; doi:10.3390/molecules28104003)
Supplement: Supplementary file 1 [file molecules-28-04003-s001.zip › molecules-2279855-supplementary.pdf]

Tuning the Site-to-Site Interaction of Heteronuclear Diatom Catalysts MoTM /C_2N (TM = 3d transition metal) for Electrochemical Ammonia Synthesis

Xiaoli Yang [1,2], Ping An[1], Ruiying Wang[1] and Jianfeng Jia [1,*]

[1] Key Laboratory of Magnetic Molecules and Magnetic Information Materials (Ministry of Education), School of Chemistry and Material Science, Shanxi Normal University, Taiyuan 030031, China

[2] Department of Pharmacy, Changzhi Medical College, Changzhi 046000, China

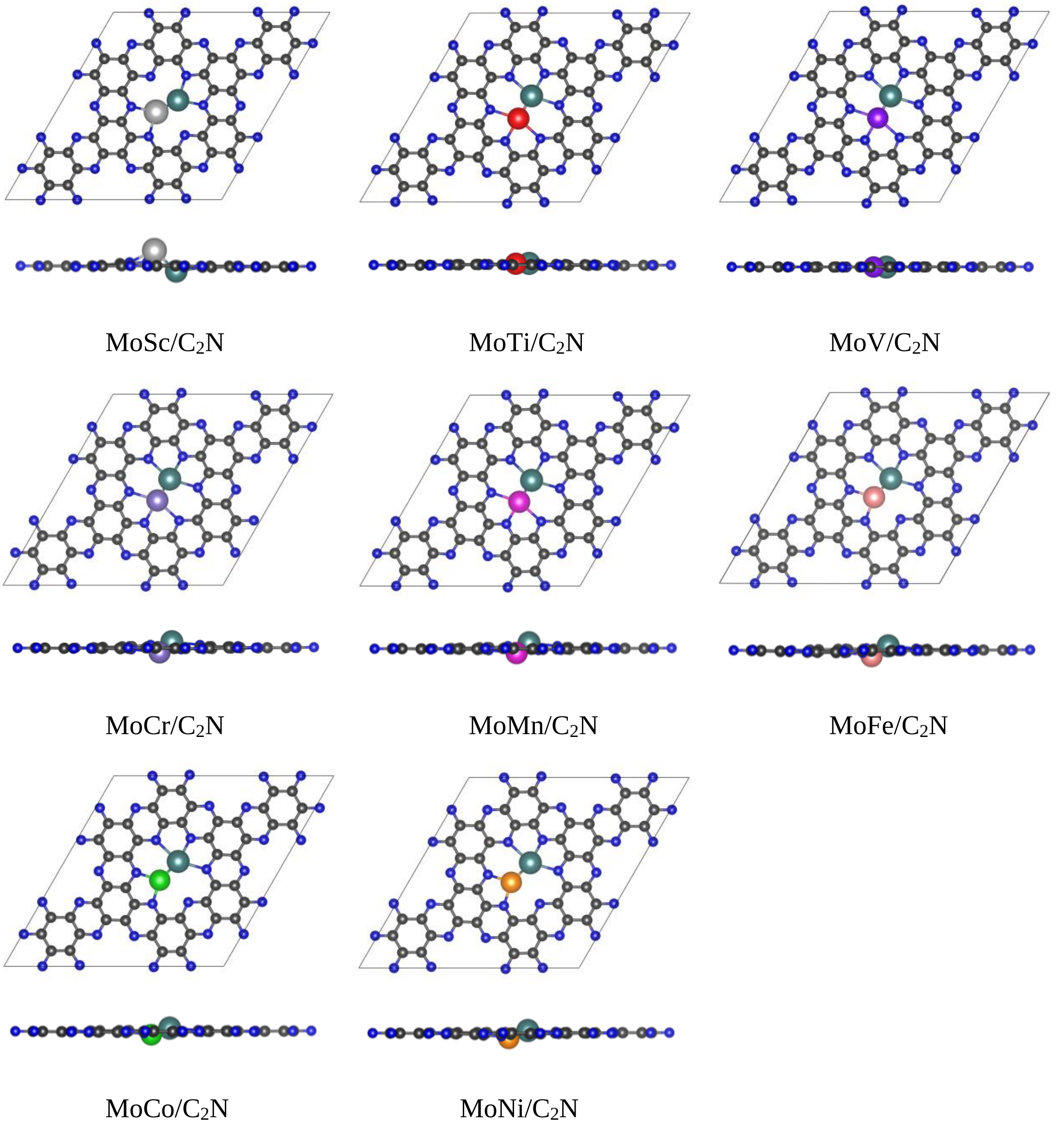

Figure S1 Optimized structures of the most stable MoTM/C_2N (TM = 3d transition metal). The C, N and Mo atoms are labeled as gray, blue and teal balls, respectively.

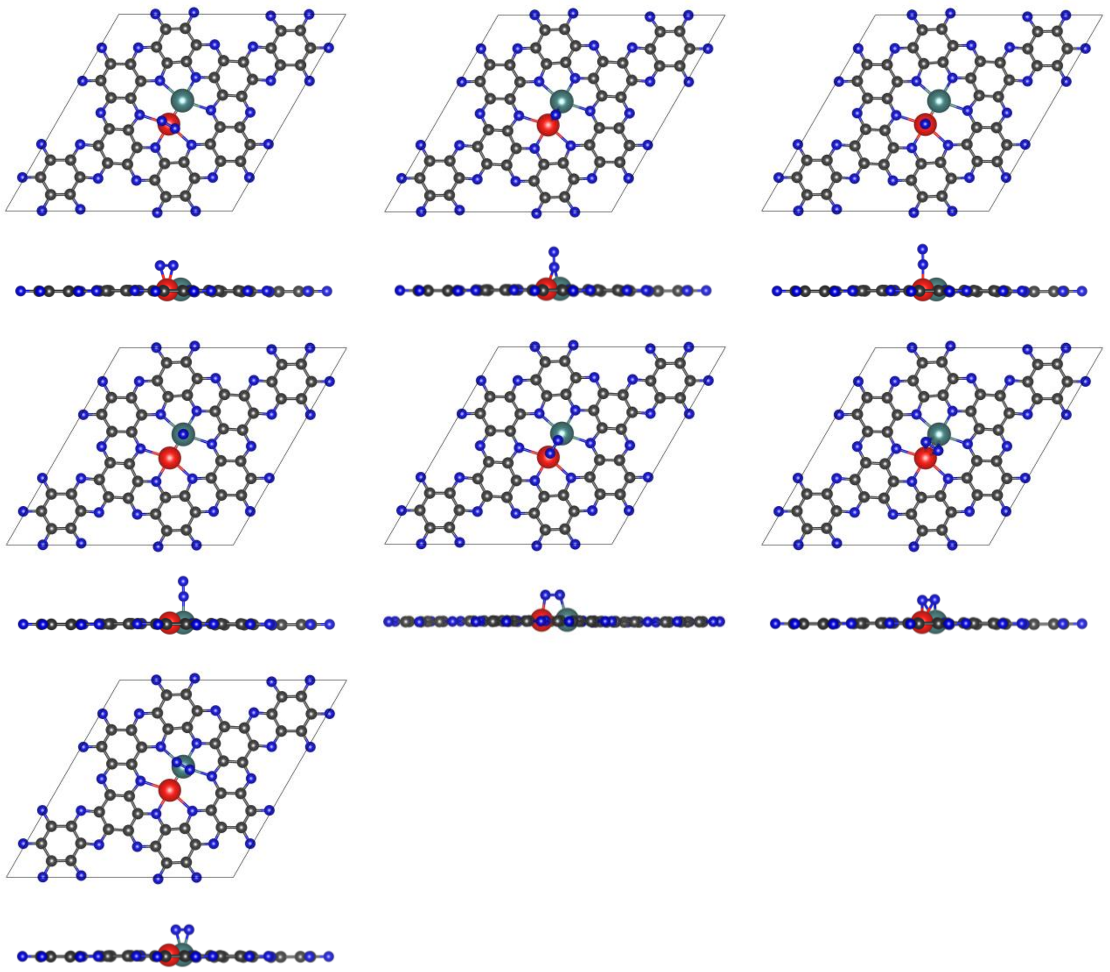

Figure S2 Possible adsorption configurations diagram of N_2 on MoTM/C_2N catalysts (take MoTi/C_2N as an example). The C, N, Mo and Ti atoms are labeled as gray, blue, teal and red balls, respectively.

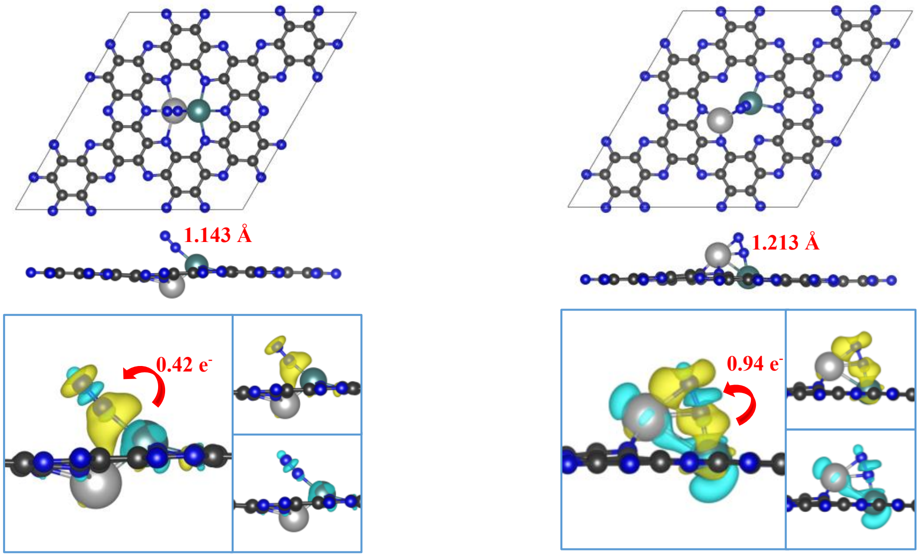

MoSc/C_2N

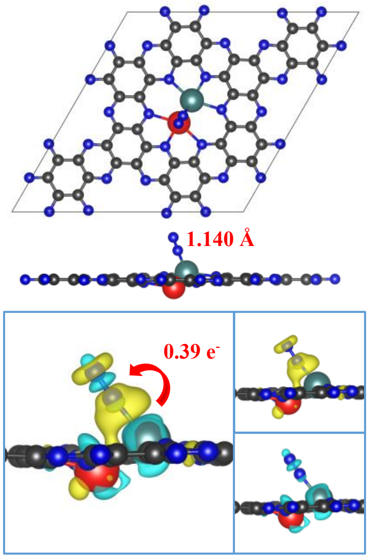

MoTi/C_2N

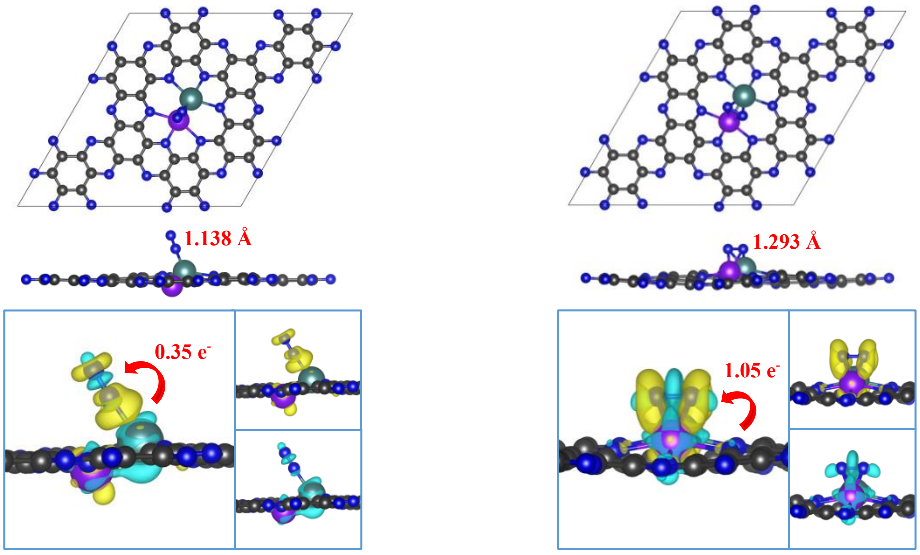

MoV/C_2N

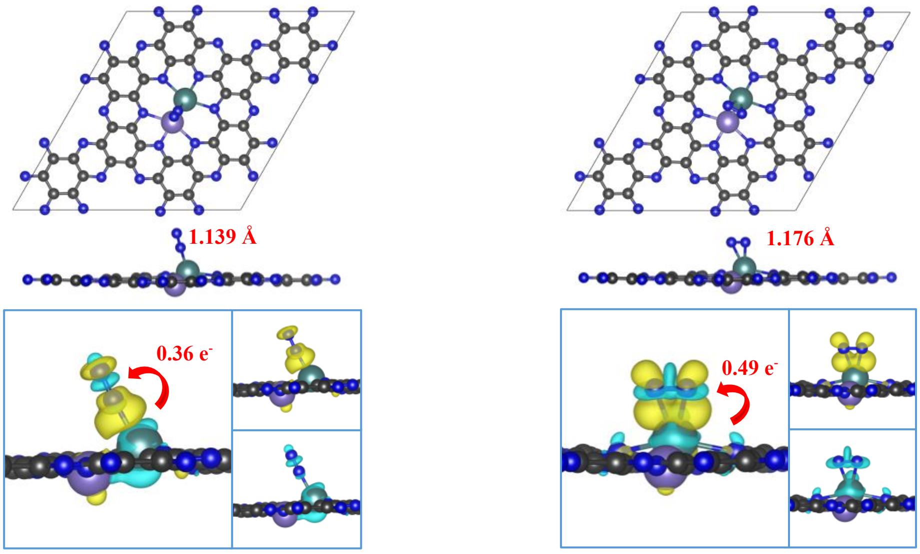

MoCr/C_2N

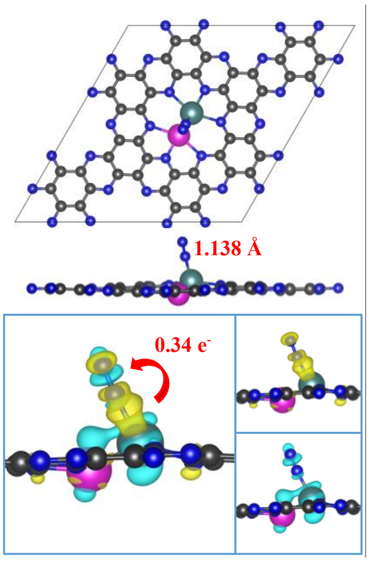

MoMn/C_2N

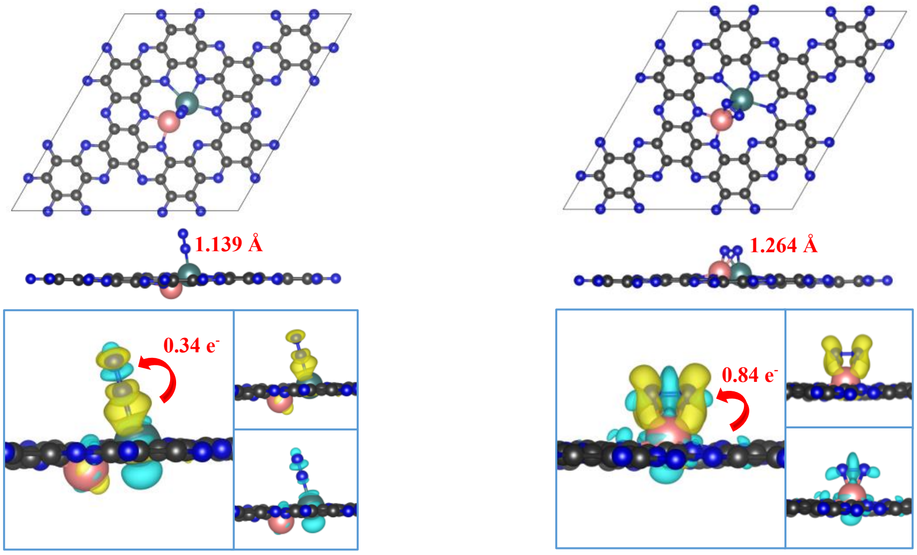

MoFe/C_2N

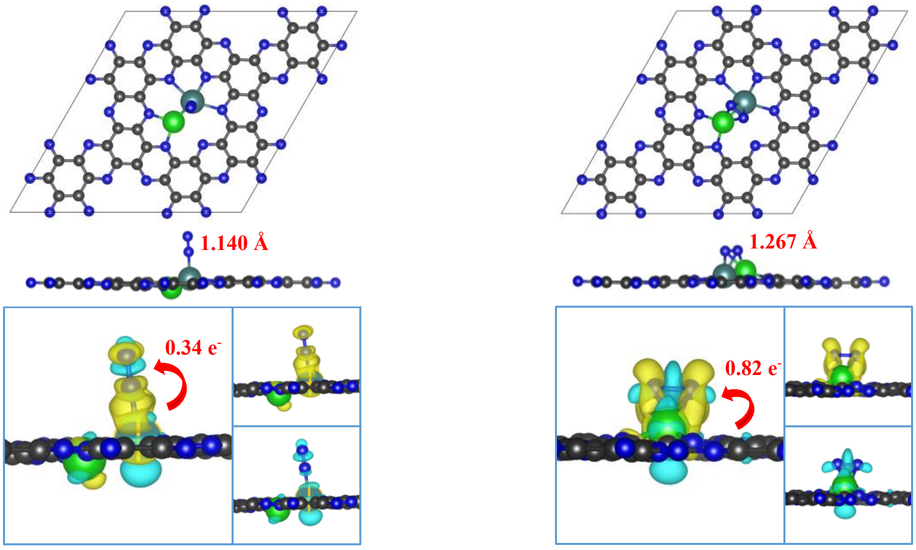

MoCo/C_2N

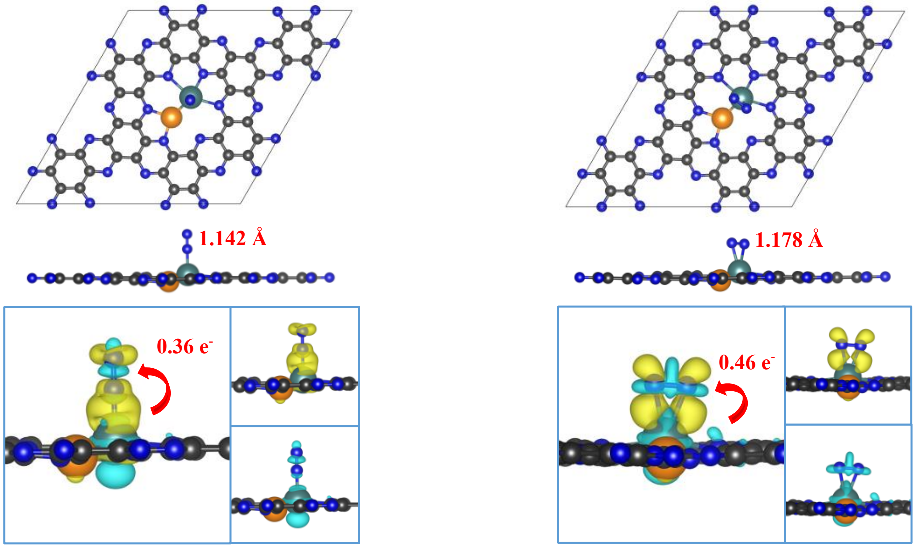

MoNi/C_2N

Figure S3 Optimized adsorption configurations and charge density differences of N_2 chemisorbed on MoTM/C_2N (TM = 3d transition metal). The charge accumulation and depletion were depicted by yellow and cyan, respectively. The isosurface value is 0.003 e/Å^3.

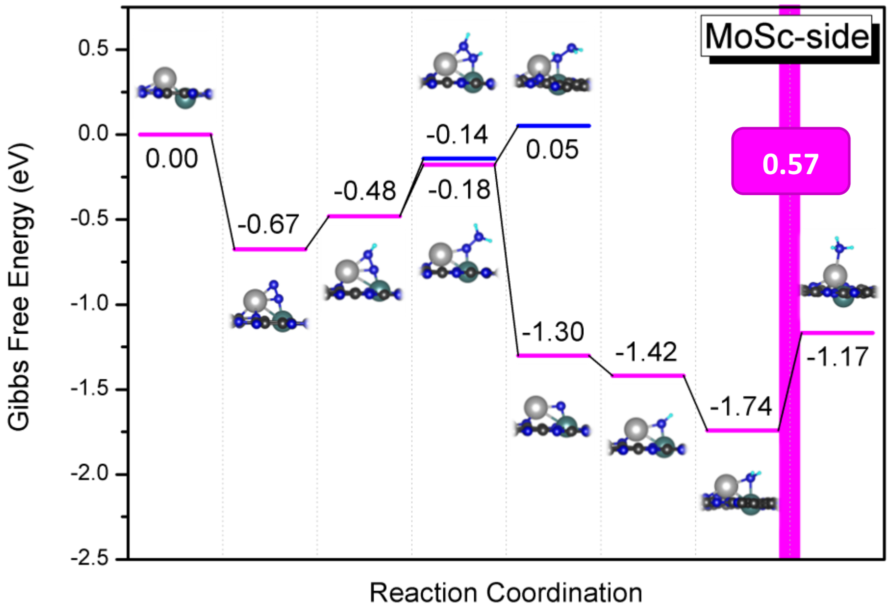

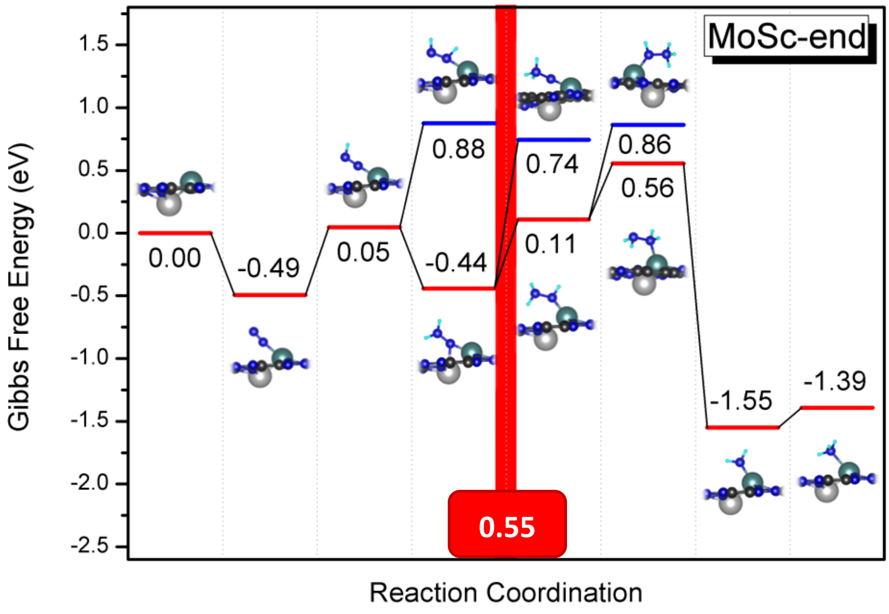

Figure S4 Gibbs free energy diagrams for NRR on MoSc/C_2N.

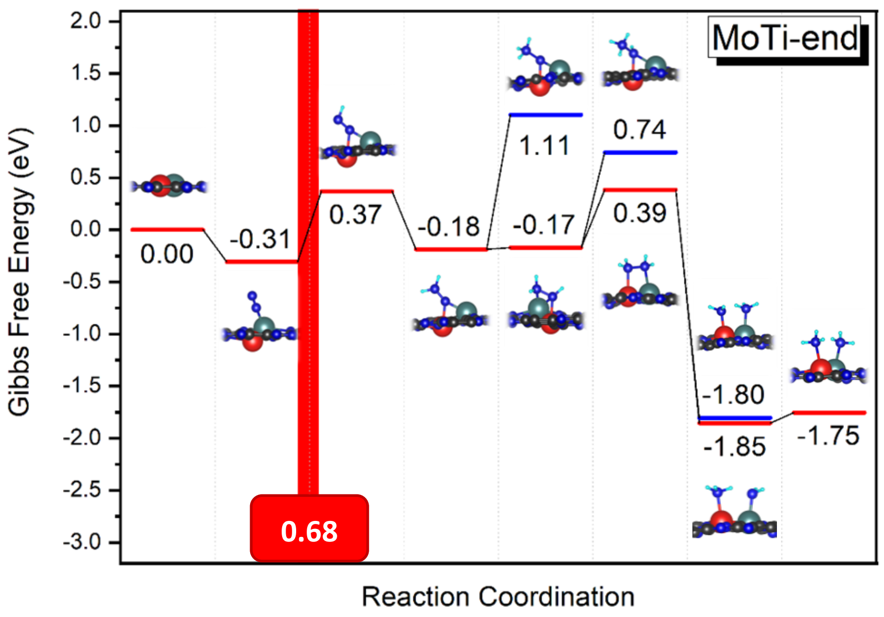

Figure S5 Gibbs free energy diagram for NRR on MoTi/C_2N.

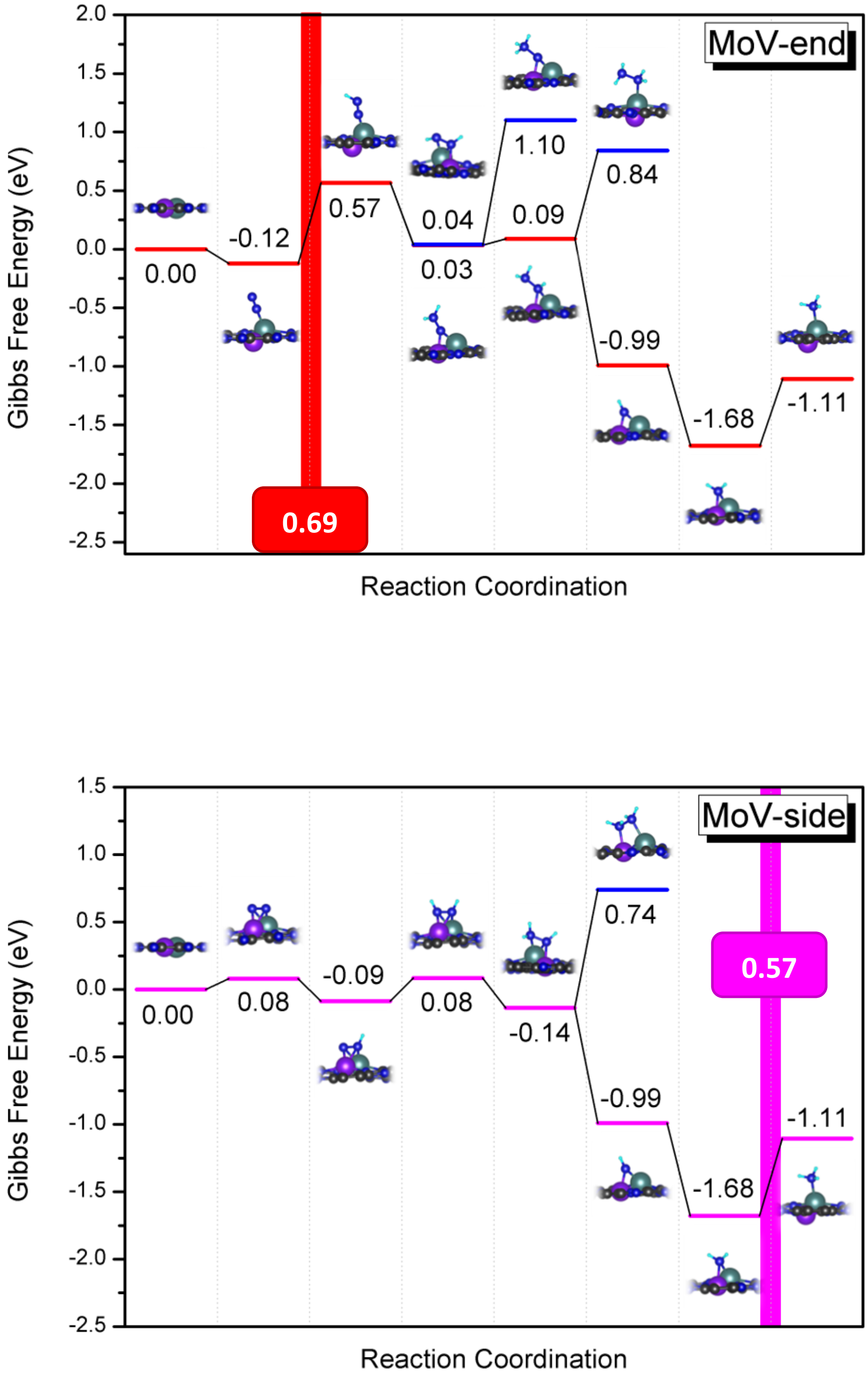

Figure S6 Gibbs free energy diagrams for NRR on MoV/C_2N.

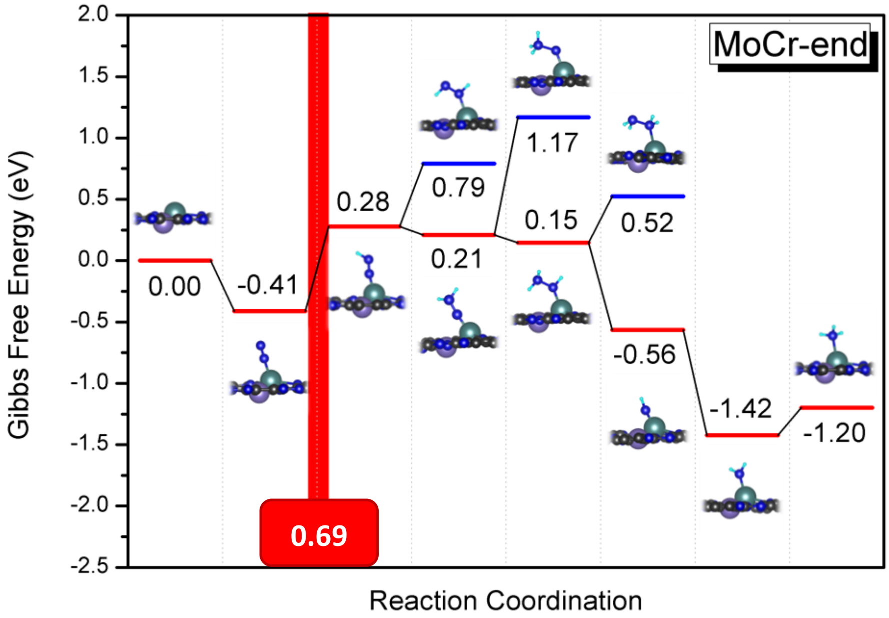

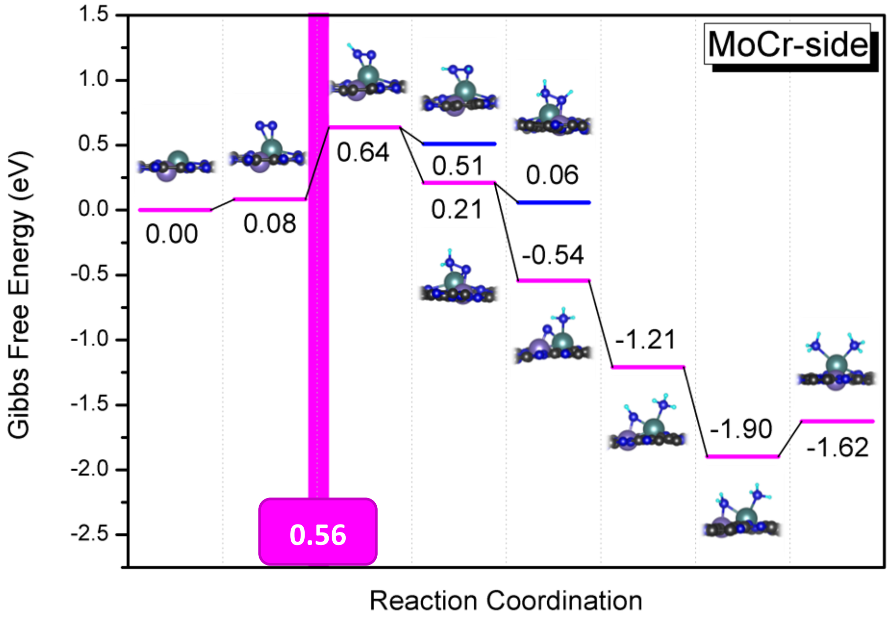

Figure S7 Gibbs free energy diagrams for NRR on MoCr/C_2N.

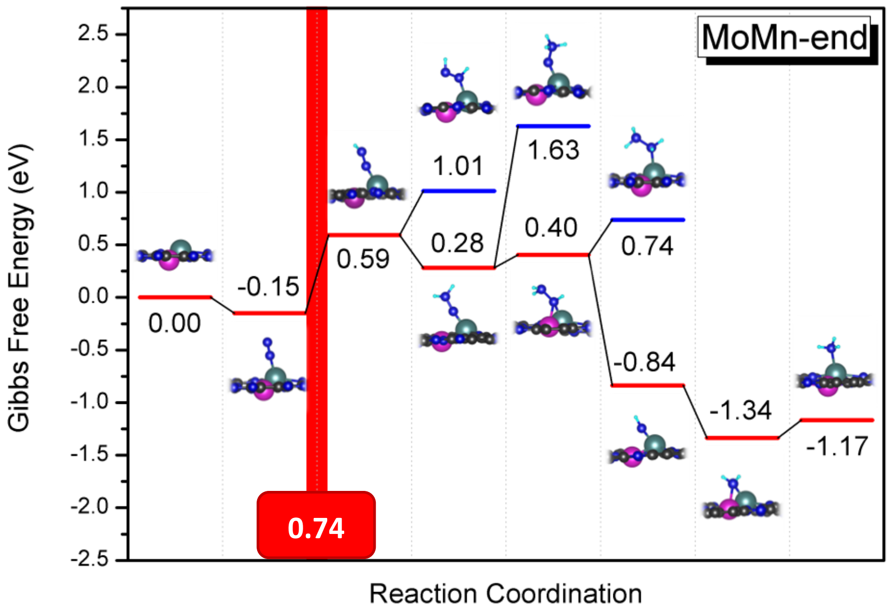

Figure S8 Gibbs free energy diagram for NRR on MoMn/C_2N.

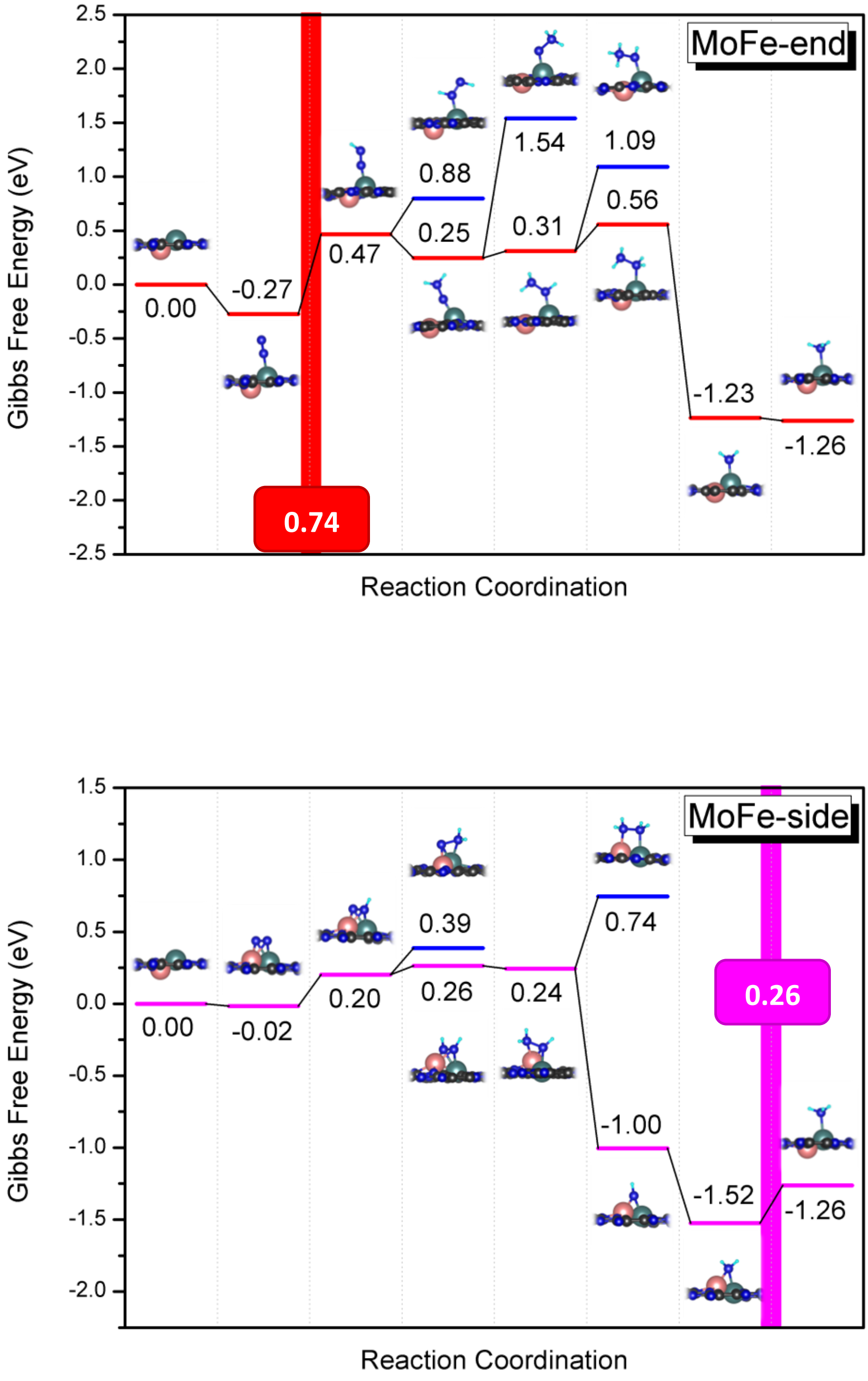

Figure S9 Gibbs free energy diagrams for NRR on MoFe/C_2N.

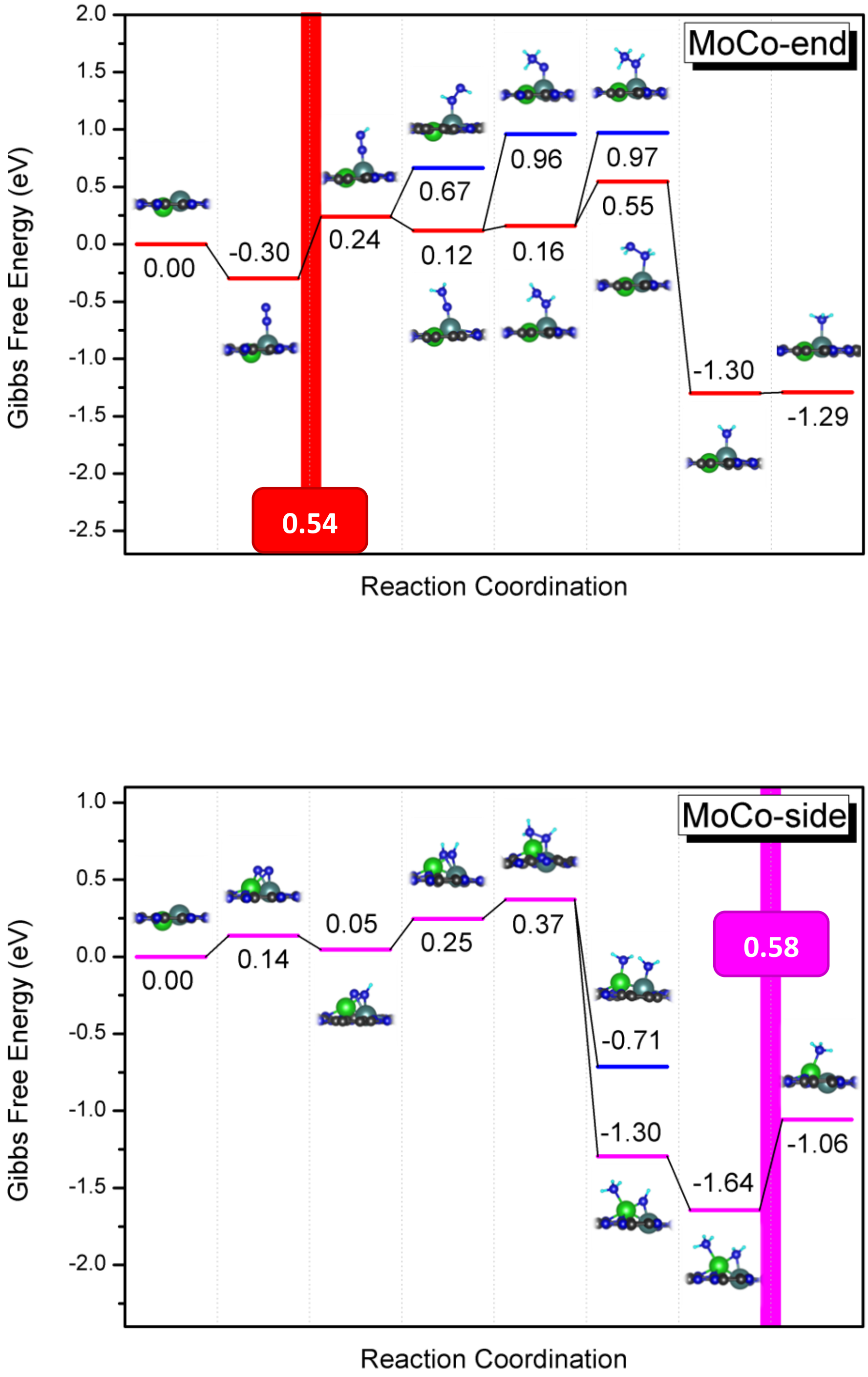

Figure S10 Gibbs free energy diagrams for NRR on MoCo/C_2N.

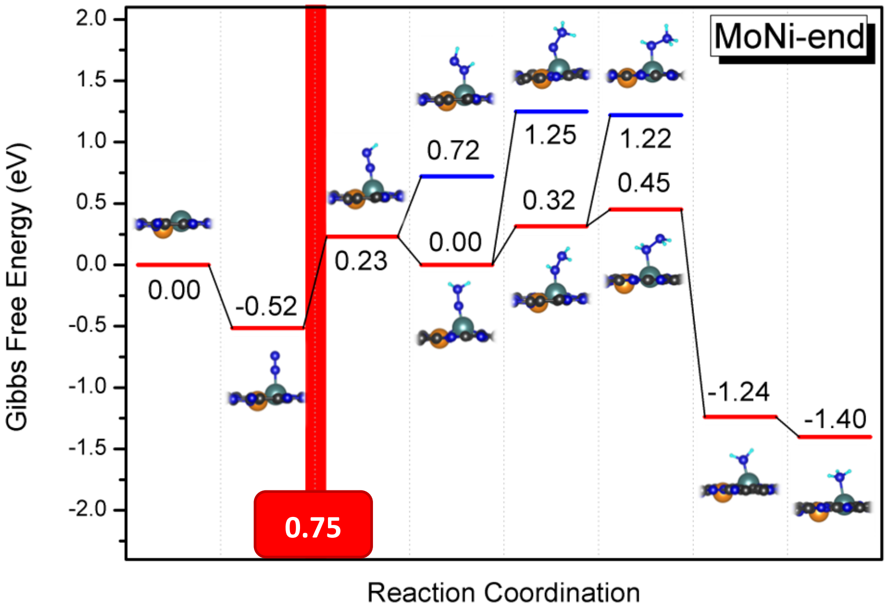

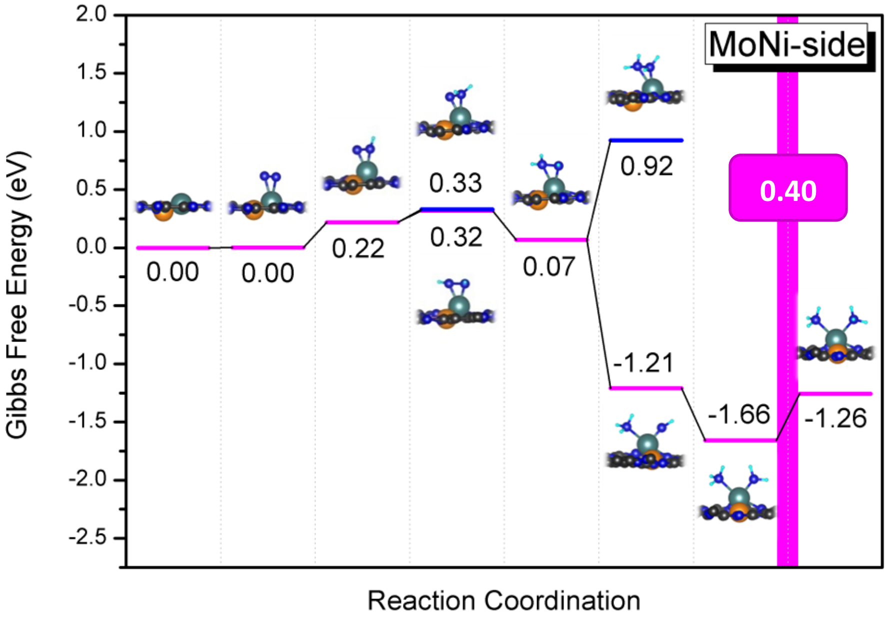

Figure S11 Gibbs free energy diagrams for NRR on MoNi/C_2N.

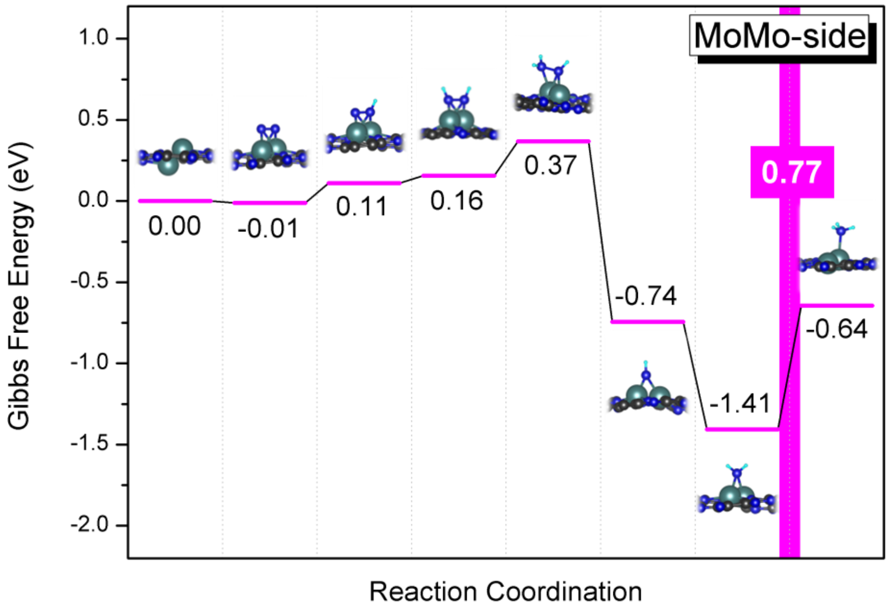

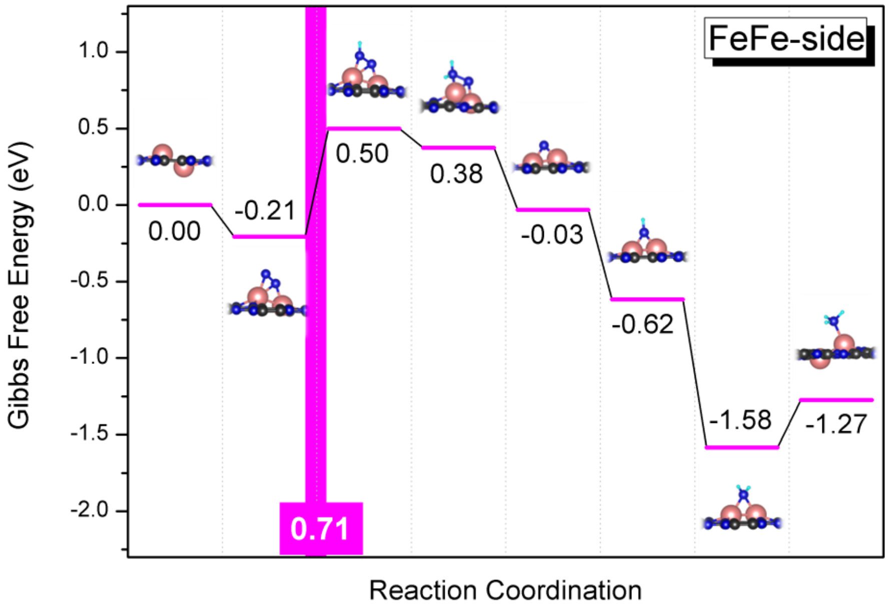

Figure S12 Gibbs free energy diagrams for NRR on MoMo/C_2N and FeFe/C_2N, respectively.

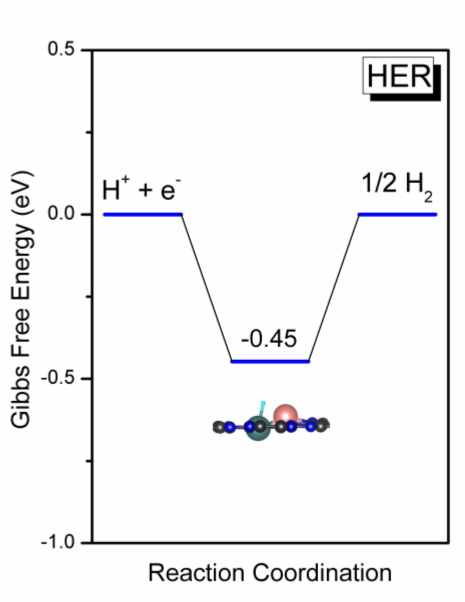

Figure S13 Gibbs free energy diagram of HER on MoFe/C_2N.

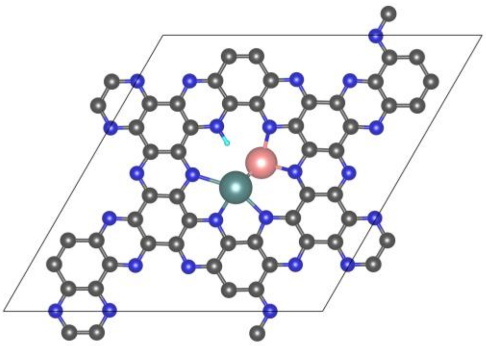

Figure S14 The *H adsorption on the N site of MoFe/C_2N.

Table S1. The bond length of Mo-TM (d_{Mo-TM}), the binding energy (E_b) of MoTM and the atomic charge of anchored Mo and TM in the system of MoTM/C_2N (TM = 3d transition metal), and the bond length of TM-TM in their bulk phase (D_{TM-TM}).

	d_{Mo-TM} (Å)	D_{TM-TM} (Å)	the binding energy of MoTM (eV)	the atomic charge of anchored Mo |e|	the atomic charge of anchored TM |e|
MoSc/C_2N	2.48	3.22	-10.53	+0.78	+2.32
MoTi/C_2N	1.99	2.98	-11.51	+0.66	+1.08
MoV/C_2N	1.93	2.58	-10.98	+0.86	+0.96
MoCr/C_2N	2.04	2.46	-9.10	+0.94	+0.88
MoMn/C_2N	2.03	2.47	-9.15	+0.99	+0.68
MoFe/C_2N	2.04	2.43	-9.68	+1.09	+0.61
MoCo/C_2N	2.05	2.48	-9.88	+1.12	+0.46
MoNi/C_2N	2.15	2.49	-10.64	+1.16	+0.43
MoMo/C_2N	2.13	2.74	-10.88	+0.93	+0.93
FeFe/C_2N	2.22	2.43	-8.51	+0.77	+0.77
